# Diagnostic performance of calculated serum osmolarity versus clinical impression for identifying dehydration in older hospitalized patients

**DOI:** 10.1186/s12877-026-08503-6

**Published:** 2026-10-16

**Authors:** Maryam Pourhassan, Lukas Funk, Maria Schnepper, Isabel Levermann, Baigang Wang, Chantal Giehl, Ulrike Trampisch, Rainer Wirth

**Affiliations:** Department of Geriatric Medicine, Marien Hospital Herne, Ruhr-Universität Bochum, Herne, Germany

**Keywords:** Dehydration, Older adults, Calculated osmolarity, Glucose, Osmolality

## Abstract

**Objectives:**

This study aimed to evaluate the diagnostic performance and agreement of calculated serum osmolarity for identifying dehydration in older hospitalized patients, using clinical impression of dehydration as the reference standard, and to examine glucose levels across concordant and discordant classification groups.

**Design and setting:**

This prospective, observational study was conducted in a geriatric acute care unit.

**Participants:**

The study included 455 older hospitalized patients with a mean age of 82.2 ± 6.8 years and 70% were female.

**Measurements:**

Clinical impression of dehydration (dehydration vs. no dehydration [normal hydration + hyperhydration]) was judged and recorded by the attending physician within 24 h of admission. Calculated serum osmolarity was derived from routine laboratory values and classified as high (≥ 295 mOsmol/l) or normal (< 295 mOsmol/l). Diagnostic accuracy (sensitivity, specificity, predictive values, accuracy) and agreement (Cohen’s κ) were calculated. Glucose was compared between concordant/discordant groups.

**Results:**

Clinically, 23% of patients were classified as dehydrated. High calculated osmolarity occurred in 38%. Using the ≥ 295 mOsmol/l cut-off, sensitivity was 48.6% and specificity 65.1%; positive and negative predictive values were 29.7% and 80.7%, respectively, and accuracy was 61.3%. Agreement beyond chance was poor (κ = 0.112). Mean glucose was higher in osmolarity-positive groups, including false positives (all *p* < 0.001 for planned pairwise comparisons).

**Conclusion:**

In older hospitalized patients, calculated serum osmolarity (≥ 295 mOsmol/l) showed limited agreement with clinical dehydration assessment with frequent osmolarity-positive classifications in clinically non-dehydrated patients. These findings support cautious clinical interpretation of calculated osmolarity in acute care, particularly when glucose is elevated.

**Supplementary information:**

The online version contains supplementary material available at https://doi.org/10.1186/s12877-026-08503-6.

## Introduction

There is no universally accepted definition of dehydration, and assessment of hydration status remains complex. Dehydration can be considered a broad clinical term describing a clinically relevant deficit of body fluid, irrespective of the underlying aetiology, osmotic or electrolyte pattern [1]. Different forms of fluid deficit may occur, including hypertonic low-intake dehydration and volume depletion. These conditions may coexist but represent different physiological disturbances and should therefore be distinguished when interpreting clinical and laboratory findings. Low-intake dehydration is the most frequent form in older adults which results from insufficient fluid intake relative to fluid losses and typically leads to a hypertonic state with increased serum osmolality [1]. All forms of dehydration may be associated with a broad spectrum of clinical symptoms and functional limitations [2]. Age-related physiological changes, including reduced thirst perception, together with polypharmacy, cognitive impairment, and impaired access to fluids increase susceptibility to low-intake dehydration [3, 4]. Dehydration has been associated with adverse outcomes in older adults, including falls, cardiovascular complications, and delirium, and has repeatedly been linked to increased morbidity and mortality [4–9]. Timely recognition is therefore clinically important, yet remains challenging in routine practice.

Dehydration is commonly assessed clinically using information from the patient history and physical examination. However, in older patients, clinical signs and symptoms are often non-specific, and there is no universally accepted single gold standard for diagnosing dehydration. Importantly, several bedside assessment methods frequently used in practice, such as skin turgor, urine colour and volume, heart rate, dry mouth, and thirst sensation, have limited reliability for detecting low-intake dehydration in older adults [2]. In addition, clinical signs that may be suggestive of dehydration (e.g., tongue furrows, dry mucous membranes, or increased urine specific gravity) can be influenced by comorbidities and medications that are common in this age group, which may lead to misclassification [10]. Given the limited diagnostic value of individual signs, clinical assessment should be based on an overall judgement that integrates symptoms, examination findings, response to therapy, and available clinical information. Finally, the therapy of acute dehydration is rather simple, however, evidence for effective prevention strategies remains limited, and the optimal daily fluid intake for older adults is not well established [11].

While different types of dehydration exist, low-intake dehydration is considered the most common form in older adults, leading to hypertonic dehydration [11]. The diagnosis of this type of dehydration may also be supported by laboratory assessment and is therefore frequently used to complement clinical decision-making. Serum osmolality is most commonly applied, with values > 300 mOsm/kg often considered suggestive of dehydration [12]. Serum osmolality can be measured directly using an osmometer (e.g., freezing-point depression), providing an estimate of the total concentration of osmotically active particles in serum/plasma [13]. The ESPEN guideline on clinical nutrition and hydration in geriatrics recommends that, when direct measurement is not available, osmolarity may be calculated from routinely measured osmotically active constituents (e.g., sodium, glucose, and urea) [11]. This approach is widely used as a pragmatic surrogate marker and is commonly interpreted using thresholds such as ≥ 295 mOsmol/l to indicate dehydration [1, 14].

However, an important limitation of both measured osmolality and calculated osmolarity is that it reflects osmotic concentration rather than hydration status per se. Osmolality can be increased by conditions that raise osmotically active solutes independent of fluid deficit, such as hyperglycaemia, elevated urea in renal impairment, or other disturbances of electrolyte balance [13, 15]. Consequently, elevated osmolality in these clinical situations may not directly indicate low-intake dehydration, and calculated osmolarity in particular may be disproportionately influenced by high glucose values [16]. Despite its practical advantages, the extent to which calculated osmolarity aligns with clinicians’ bedside classification of dehydration in older inpatients is uncertain. Improved understanding of agreement and diagnostic performance is needed to inform how calculated osmolarity should be interpreted in geriatric inpatient care.

Therefore, this study aimed to determine how well calculated serum osmolarity (using a cut-off of ≥ 295 mOsmol/l) identifies dehydration in older hospitalised patients when compared with the clinical impression of dehydration as the reference standard. Specifically, we aimed to: (i) assess agreement between clinical dehydration status and calculated osmolarity (ii) quantify diagnostic performance (sensitivity, specificity, positive predictive value, negative predictive value, and accuracy), and (iii) describe glucose levels according to concordant versus discordant classifications between clinical impression and calculated osmolarity.

## Subjects and methods

### Study population

This study is a secondary cross-sectional analysis of prospectively collected data from a larger multicentre, observational study conducted between December 2022 and March 2024, which aimed to investigate causes of protein-energy malnutrition in older adults in both inpatient and outpatient settings. The present study is reported in accordance with the STROBE guidelines for cross-sectional observational studies (Supplementary File 1). Results from the outpatient component of the original study have been published [17], whereas the inpatient component is currently under review. Within the inpatient cohort, we additionally captured clinical impression of dehydration and edema and evaluated the agreement between clinical impression of dehydration (reference standard) and calculated serum osmolarity. The sample size calculation was performed for the primary objective of the original study and not for the present secondary analysis.

Participants of this analysis were exclusively enrolled at Marien Hospital Herne (*n* = 455), because laboratory parameter were only available in these participants. Eligibility criteria included age ≥ 75 years and availability of a valid weight measurement. Exclusion criteria were terminal illness; severe disturbances of fluid balance (e.g., decompensated heart failure or clinically obvious symptomatic dehydration); requirement for haemodialysis or parenteral nutrition for more than two weeks; and limb amputation.

### Comprehensive geriatric assessment and further trial measurements

All patients were evaluated by a comprehensive geriatric assessment (CGA) at time of hospital admission assessing frailty, functional dependence, cognitive status, mood, nutritional status, and calf circumference. Furthermore, routine laboratory assessment, and calculated serum osmolarity, was performed.

Frailty was assessed with the Clinical Frailty Scale [18] which combines descriptive texts and pictograms to illustrate a patient’s functional and activity level, rated from 1 (very fit) to 9 (terminally ill). Functional dependence was assessed by Barthel Index that evaluates the patient’s ability to carry out self-care tasks. 40–55/100 points correspond to a moderate functional dependence, whereas 20–35/100 points represent a severe, and < 20 points a very severe functional dependence [19].

Cognitive function was assessed by the Montreal Cognitive Assessment (MoCA) test [20] and a total score below 26/30 points assumes a cognitive impairment (depending on age, sex and the level of education). Depression in Old Age Scale (DiA-S) was performed to investigate depressive symptoms and subjects were categorized as having no depression (0–2 points), suspected depression (3 point) and probable depression (4–10 points) [21].

### Definition of clinical impression of dehydration and calculated osmolarity

#### Clinical impression of dehydration

Clinical impression of dehydration is typically based on a combination of patient history and physical examination, and no single universally accepted gold standard exists. In this study, clinical impression of dehydration was assessed and recorded prospectively by the attending physician during the initial assessment period within 24 h of admission, and was used as the reference standard for all diagnostic comparisons. In this study, dehydration was defined broadly to include any clinically relevant body-fluid deficit, rather than being limited to hypertonic low-intake dehydration. Within the assessment, the attending physician evaluated all available routine data, i.e. the complete clinical picture, including anamnesis, physical examination findings such as dryness of skin and mucosa, venous filling, edema and if available ultrasound of inferior vena cava [22, 23]. For the primary analyses, patients were dichotomized as dehydration versus no dehydration, with the latter comprising normal hydration and hyperhydration. The attending physicians had also access to routine laboratory information available in clinical practice, including calculated osmolarity. However, clinical dehydration was assessed as part of the original study based on the overall clinical picture and was not defined according to a prespecified osmolarity threshold.

#### Calculated osmolarity

Although different types of dehydration exist, the present analysis focused on low-intake dehydration, which is considered the most common form in older adults [11]. Low-intake dehydration was operationalised using calculated serum osmolarity derived from routinely available laboratory values. Calculated serum osmolarity was obtained using the formula applied by the central laboratory.

Serum osmolarity (mOsmol/l) = 1.86 × (serum sodium + potassium) + 1.15 x serum glucose + serum urea + 14 (all measured in mmol/l). This formula has demonstrated best overall agreement with directly measured osmolality [13].

Patients with a calculated serum osmolarity of ≥ 295 mOsmol/l were classified as dehydrated, indicating a state of free water deficit [1, 11]. As mentioned above, patients with clinically obvious, symptomatic severe dehydration were excluded from study participation to avoid potential confounding with regard to the aim of the primary analysis, characterising malnutrition.

#### Laboratory parameters

Blood samples were obtained as part of routine clinical care at the time of hospital admission and analysed in the hospital’s central laboratory according to standard operating procedures. Laboratory parameters included electrolytes and renal function markers (e.g., sodium, potassium, urea, creatinine, uric acid, cystatin C), inflammatory markers (C-reactive protein), and glucose and HbA1c. No oral or intravenous rehydration was administered between blood sampling and clinical hydration assessment.

## Statistical methods

Statistical analyses were performed using IBM SPSS Statistics for Windows (IBM Corp., Armonk, NY, USA), version 29.0. Continuous variables are presented as mean ± standard deviation (SD) for approximately normally distributed data and as median (interquartile range [IQR]) for non-normally distributed data. Categorical variables are reported as counts and percentages. Between-group comparisons (no dehydration vs. dehydration) were performed using the independent-samples t test for normally distributed continuous variables, the Mann–Whitney U test for non-normally distributed continuous variables, and Pearson’s chi-square test for categorical variables.

Diagnostic performance of calculated serum osmolarity (test positive defined as ≥ 295 mOsmol/l) against the clinical reference standard was assessed by calculating sensitivity, specificity, positive predictive value (PPV), negative predictive value (NPV), and overall accuracy from 2 × 2 contingency tables. Positive and negative likelihood ratios (LR + and LR−) were additionally calculated from sensitivity and specificity. Agreement between calculated osmolarity category and clinical dehydration status was quantified using Cohen’s κ. Systematic differences in paired classifications were evaluated with McNemar’s test. In addition, Receiver operating characteristic (ROC) analysis was performed using calculated serum osmolarity as a continuous variable to assess its ability to discriminate between clinically dehydrated and non-dehydrated patients. Discriminatory ability was expressed as the area under the ROC curve (AUC) with 95% confidence intervals. All tests were two-sided, and a p value < 0.05 was considered statistically significant.

## Results

### Characteristics of study participants

Table 1 summarizes baseline characteristics stratified by clinical impression of dehydration (reference standard). Overall, participants had a mean age of 82.2 ± 6.8 years and 70% were female, representing a typical sex distribution in acute geriatric wards. Based on clinical assessment, 77% were classified as having no dehydration and 23% as dehydrated. Based on calculated osmolarity (valid *n* = 453), 281 (62%) of patients had normal osmolarity (< 295 mOsmol/l) and 172 (38%) had high osmolarity (≥ 295 mOsmol/l).

**Table 1:** Baseline characteristics of the study population stratified by clinical impression of dehydration*

Gender	All (*n* = 455)	No dehydration (*n* = 350, 77%)	Dehydrated (*n* = 105, 23%)	*P* value
Female, n (%)	316 (70)	252 (72)	64 (61)	0.040
Male, n (%)	139 (30)	98 (28)	41 (39)	
Age (y), mean ± SD	82.2 ± 6.8	81.8 ± 6.8	83.7 ± 6.8	0.012
Height (m), mean ± SD	1.65 ± 0.09	1.65 ± 0.08	1.66 ± 0.09	0.145
Body weight (kg), mean ± SD	72.6 ± 17.7	73.5 ± 17.6	69.8 ± 18.2	0.060
BMI (kg/m^2^), mean ± SD	26.5 ± 5.9	26.9 ± 6.0	25.0 ± 5.6	0.003
Previous weight loss				
No, n (%)	232 (51)	171 (49)	61 (58)	0.683
Yes, n (%)	223 (49)	179 (51)	44 (42)	
Previous weight loss (kg), mean ± SD	3.9 ± 6.3	4.1 ± 6.6	3.0 ± 5.5	
Duration of previous weight loss (m)				
< 3 months, n (%)	133 (29)	105 (30)	28 (27)	0.374
< 6 months, n (%)	30 (7)	25 (7)	5 (5)	
>6 months, n (%)	60 (13)	49 (14)	11 (11)	
*Geriatric assessment*				
MNA-SF, median (IQR)	8 (6–9)	8 (6–10)	7 (6–9)	0.002
Normal nutritional status, n (%)	20 (4)	16 (5)	4 (4)	0.017
Risk of malnutrition, n (%)	230 (50)	189 (54)	41 (39)	
Malnourished, n (%)	205 (45)	145 (41)	60 (57)	
Barthel-Index, median (IQR)	50 (40–60)	50 (45–60)	45 (35–60)	0.004
MoCA, Median (IQR)	18 (15–21)	19 (15–22)	16 (13–18)	< 0.001
DiA, Median (IQR)	3 (1–5)	3 (1–5)	4 (2–5)	0.013
Clinical Frailty Scale, median (IQR)	5 (5–6)	5 (5–6)	6 (5–6)	0.012
Frailty, n (%)	217 (48)	160 (46)	56 (53)	0.178
No Frailty, n (%)	238 (52)	189 (54)	49 (47)	

MNA-SF, Mini Nutritional Assessment Short Form; MoCA, Montreal Cognitive Assessment; DiA, Depression in old Age. Values are given as number (%), mean ± SD or median (IQR, interquartile range). *Clinical impression of dehydration= reference standard

Compared with patients with no dehydration, those judged clinically dehydrated were older and more often male. Clinically dehydrated patients also had a lower BMI, lower MNA-SF scores, and a higher proportion classified as malnourished according to MNA-SF. Functional and cognitive measures were poorer in the dehydrated group, including a lower Barthel Index, lower MoCA scores, higher DiA scores, and a slightly higher Clinical Frailty Scale. In contrast, previous weight loss and its duration did not differ meaningfully between groups, and the proportion classified as frail was similar.

### Laboratory parameters

Table 2 presents the comparison of laboratory parameters between patients with no dehydration and those classified as dehydrated according to the clinical reference standard. Clinically dehydrated patients had significantly higher urea, uric acid, creatinine, leukocyte counts, and albumin levels. LDL-cholesterol levels were significantly lower in dehydrated participants. All other laboratory measures, including calculated osmolarity did not significantly differ by hydration status.

**Table 2:** Laboratory parameters in patients with no dehydration and dehydration classified according to the clinical reference standard

	No dehydration (*n* = 350, 77%)	Dehydration (*n* = 105, 23%)	**P value
Urea (mg/dl)	43.90 ± 23.69	58.51 ± 40.85	< 0.001
Uric acid (mg/dl)	5.45 ± 2.13	6.05 ± 2.32	0.013
Cystatin C (mg/l)	3.03 ± 12.91	1.85 ± 0.93	0.615
Potassium (mmol/l)	4.26 ± 0.56	4.22 ± 0.58	0.559
Sodium (mmol/l)	138.23 ± 3.55	138.02 ± 5.43	0.707
Calcium (mmol/l)	2.27 ± 0.15	2.25 ± 0.17	0.309
Magnesium (mg/dl)	1.95 ± 0.24	1.95 ± 0.28	0.852
Phosphate (mg/dl)	3.22 ± 0.59	3.33 ± 0.81	0.173
Total cholesterol (mg/dl)	178.54 ± 51.05	169.32 ± 38.75	0.069
Triglycerides (mg/dl)	142.79 ± 68.24	149.66 ± 69.04	0.410
HDL cholesterol (mg/dl)	56.42 ± 18.10	53.30 ± 16.57	0.145
LDL cholesterol (mg/dl)	106.99 ± 51.93	96.49 ± 33.03	0.023
Folic acid (ng/dl)	7.47 ± 6.75	7.20 ± 4.69	0.739
Vitamin B12 (pg/ml)	600.00 ± 335.17	581.53 ± 329.44	0.628
Leukocytes (1000/µl)	8.34 ± 2.57	9.08 ± 3.34	0.040
Lymphocytes absolute (1000/µl)	1.51 ± 1.81	1.67 ± 1.34	0.403
Haemoglobin (g/dl)			
Male	11.79 ± 2.25	12.23 ± 2.02	0.265
Female	11.56 ± 1.87	11.31 ± 1.70	0.310
Glucose (mg/dl)	124.65 ± 41.52	130.30 ± 46.06	0.261
HbA1c (%)	5.94 ± 1.01	6.08 ± 1.00	0.250
Creatinine (mg/dl)	1.03 ± 0.44	1.28 ± 0.62	< 0.001
NT-proBNP (pg/ml)	2294.4 ± 4220.5	2653.4 ± 5265.2	0.480
Total protein (mg/l)	62.67 ± 6.59	62.43 ± 6.64	0.738
Albumin (g/dl)	3.59 ± 0.57	3.72 ± 0.57	0.048
CRP (mg/dl)	2.54 ± 3.21	2.85 ± 4.26	0.421
Calculated osmolarity (mosmol/l)	290.93 ± 9.31	293.09 ± 13.50	0.128

### Agreement between clinical dehydration and calculated osmolarity

As shown in Table 3, using the proposed cut-off of calculated osmolaliry ≥ 295 mOsmol/l, the test identified 51 of 105 patients judged clinically dehydrated, corresponding to a sensitivity of 48.6%. Among patients judged clinically not dehydrated, 226 of 347 were correctly classified as test-negative (specificity 65.1%). The corresponding positive and negative likelihood ratios were 1.39 and 0.79, respectively.

**Table 3:** Agreement between clinical impression of dehydration (reference standard) and calculated osmolality category

		Clinically dehydrated	Clinically not dehydrated	Total
Calculated osmolarity	≥295 (mOsmol/l)	51 (TP)	121 (FP)	172
	<295 (mOsmol/l)	54 (FN)	226 (TN)	280
	Total	105	347	452
Diagnostic performance	Sensitivity	48.6% (51/105)		
	Specificity	65.1% (226/347)		
	PPV	29.7% (51/172)		
	NPV	80.7% (226/280)		
	Accuracy	61.3% (277/452)		
	LR+	1.39		
	LR**−**	0.79		
Agreement / discordance	Cohen’s κ	0.112 (*p* = 0.011)		
	McNemar test	*p* < 0.001		

TP=true positive; FN=false negative; FP=false positive; TN=true negative; PPV=positive predictive value; NPV=negative predictive value; LR+, Positive likelihood ratio; LR-, Negative likelihood ratio. Test positive defined as calculated osmolarity ≥ 295 mOsmol/l; reference standard was clinical impression of dehydration

The positive predictive value was low (29.7%), indicating that most patients with calculated osmolarity ≥ 295 mOsmol/l were not clinically judged dehydrated (121/172 false positives). In contrast, the negative predictive value was higher (80.7%), suggesting that calculated osmolarity < 295 mOsmol/l was generally consistent with the absence of clinical dehydration. Overall accuracy was 61.3%. Agreement beyond chance was slight (Cohen’s κ = 0.112, *p* = 0.011).

**Fig. 1:**
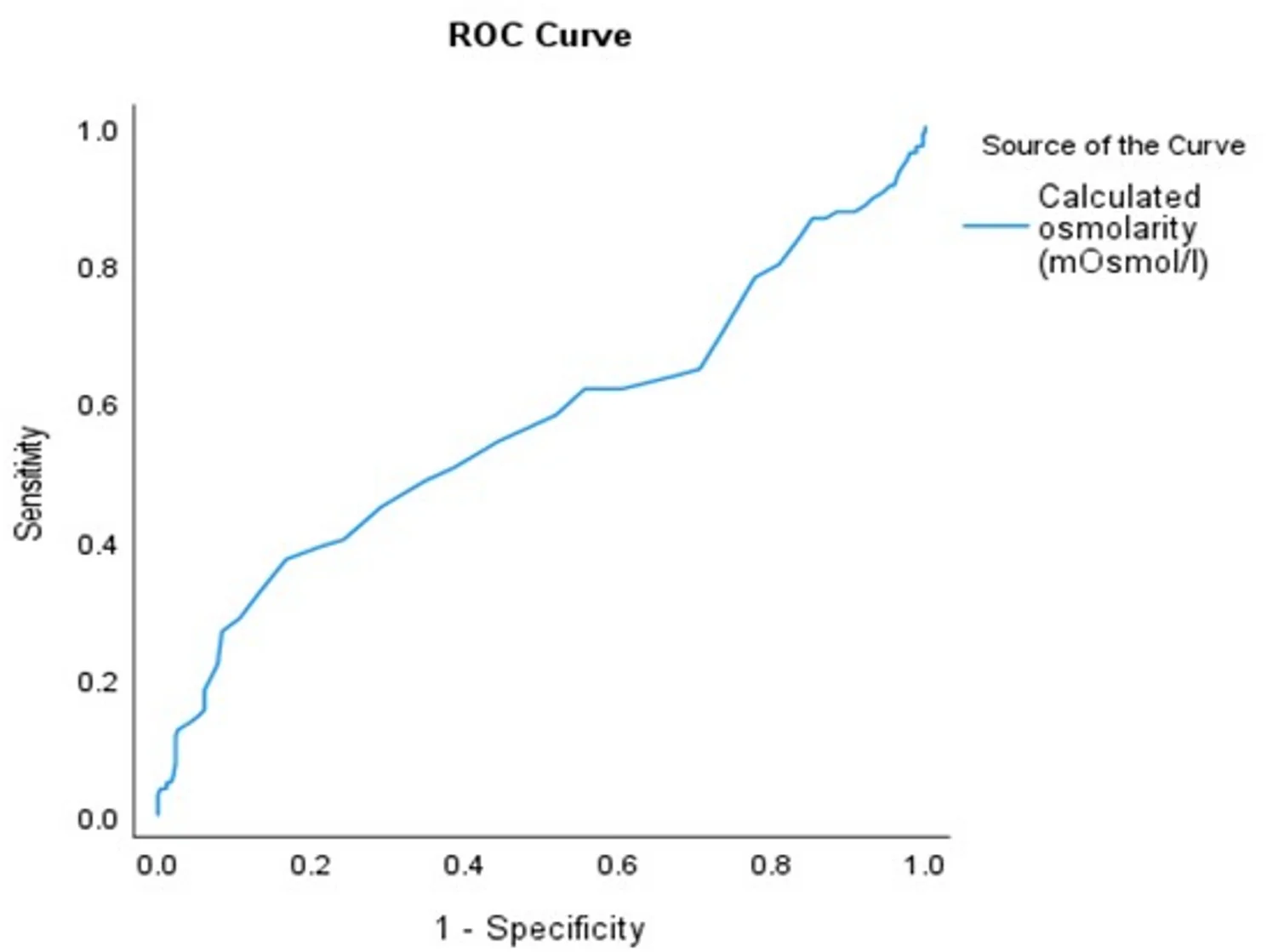
Receiver operating characteristic (ROC) curve of calculated osmolarity (mOsmol/l) for identifying clinical dehydration

The McNemar test was significant (*p* < 0.001), demonstrating systematic disagreement between calculated osmolarity and the clinical reference standard, driven primarily by an excess of false positives relative to false negatives. ROC analysis also showed limited discrimination between clinically dehydrated and non-dehydrated patients (AUC = 0.567, 95% CI 0.497–0.637; Fig. 1).

### Glucose distribution across concordant and discordant classification groups

**Fig. 2:**
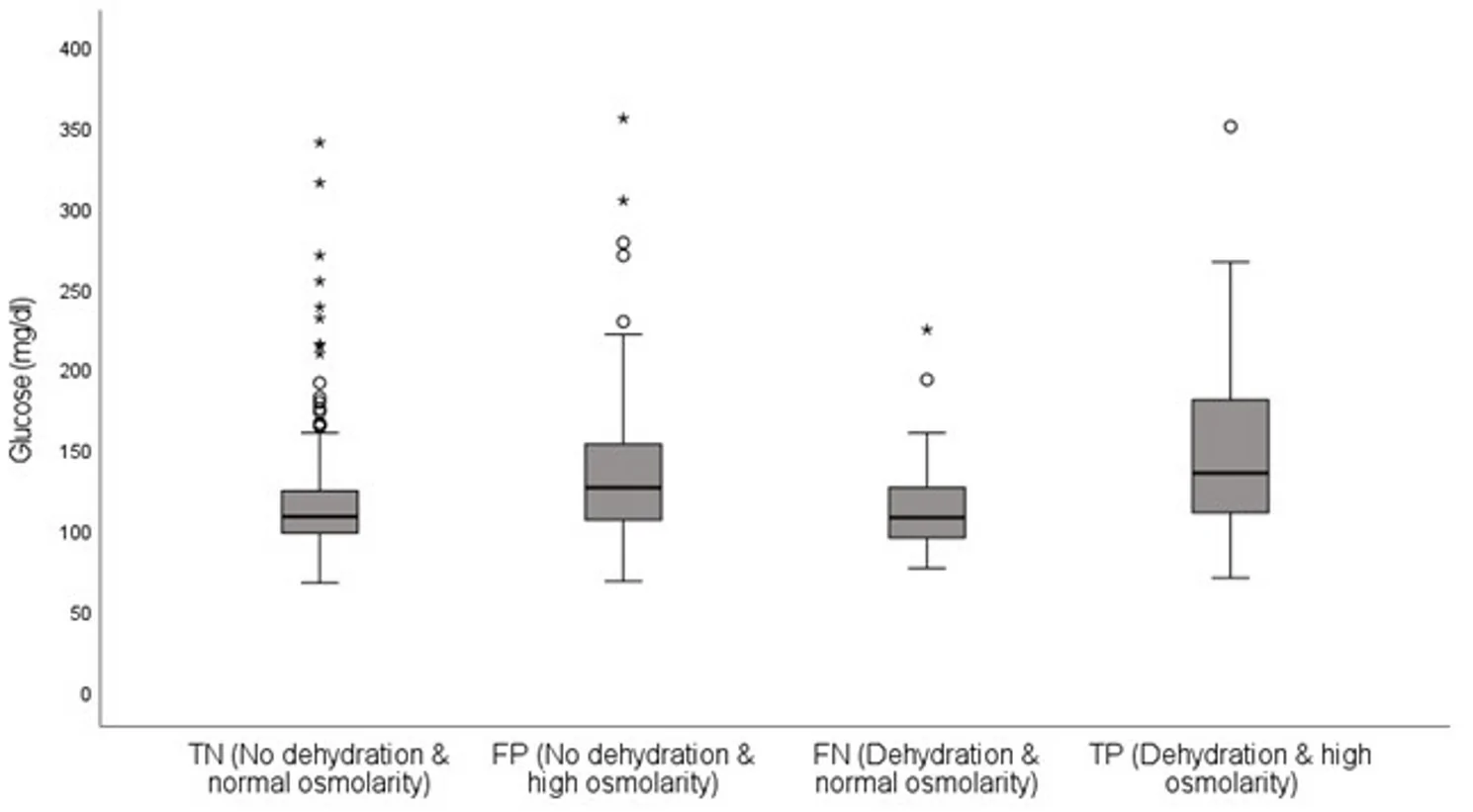
Glucose concentrations by concordance/discordance between clinical dehydration status and calculated osmolarity category. Boxplots show glucose (mg/dl) in four groups defined by clinical dehydration (reference standard) and calculated osmolarity (< 295 vs. ≥ 295 mosmol/l): True Negatives (TN: no clinical dehydration & osmolarity < 295 mosmol/l), False Positives (FP: no clinical dehydration & osmolarity ≥ 295 mosmol/l), False Negatives (FN: clinical dehydration & osmolarity < 295 mosmol/l), and True Positives (TP: clinical dehydration & osmolarity ≥ 295 mosmol/l). Boxes indicate median and interquartile range; Groups differed (Kruskal–Wallis *p* < 0.001)

Figure 2 shows the distribution of glucose concentrations across the four classification groups defined by clinical dehydration status and calculated osmolarity category. Median glucose levels are higher in the osmolarity-positive groups (false positives and true positives) than in the osmolarity-negative groups (true negatives and false negatives), suggesting that elevated glucose co-occurred with higher calculated osmolarity. In pairwise comparisons, mean glucose was higher in false positives than in true negatives (137.07 vs. 118.02 mg/dl, *p* < 0.001) and higher in true positives than in false negatives (149.51 vs. 112.17 mg/dl, *p* < 0.001).

## Discussion

In this cohort of older hospitalised patients, calculated serum osmolarity showed limited agreement with the clinical impression of dehydration. Sensitivity, specificity and positive predictive value were also low when clinical impression was used as the reference standard. As a result, many patients classified as osmolarity-positive were not judged clinically dehydrated. During a previous analysis with similar results [24], we noticed that discordance between clinical impression and osmolarity was sometimes associated with elevated serum glucose. Therefore, we systematically compared glucose levels across concordant and discordant classification groups in the present analysis. In our study, mean glucose levels were similar between clinically dehydrated and non-dehydrated patients. However, differences were seen when patients were grouped according to agreement between clinical impression and calculated osmolarity. Interestingly, glucose concentrations were significantly higher in osmolarity-positive patients, including those classified as false positives, suggesting that osmolarity-based positivity frequently occurred in the presence of elevated glucose. However, although the selected formula has been reported to show the best agreement with directly measured osmolality [13], it remains unclear whether it aligns well with the clinical diagnosis of dehydration. In acutely ill older hospitalized patients, where elevated glucose levels are common, calculated osmolarity may be less suitable as a stand-alone marker to support the clinical diagnosis of dehydration.

In routine care, dehydration is usually diagnosed based on an overall clinical impression that combines the patient’s history, physical examination, and the clinical context, including comorbidities and medications. However, in older adults, particularly in acute hospital settings, single signs and symptoms often have limited diagnostic value and may be influenced by multimorbidity, polypharmacy, cognitive impairment, and acute illness, which reduces the reliability of bedside assessment. Reviews focusing on older patients have reported that many commonly used clinical indicators of hydration status are not consistently reliable [2]. In addition, the lack of a universally accepted gold standard and the use of different definitions and reference methods make studies difficult to compare and limit the translation of research findings into practical recommendations for everyday clinical care.

In line with the ESPEN guideline, directly measured serum/plasma osmolality is proposed as the preferred marker for low-intake dehydration, while calculated osmolarity (including sodium/potassium, glucose, and urea) with an action threshold > 295 mmol/l for screening is suggested as a pragmatic alternative when direct measurement is not available [14]. However, according to the ESPEN guideline, interpreting a raised osmolarity as dehydration requires considering whether glucose (and to some extent urea) is within the normal range, because elevations in these osmotically active solutes can increase osmolarity independently of low-intake dehydration, an effect also reflected in our data. Our findings do not introduce a new mechanism, but provide clinical data on the extent to which this known limitation affects agreement between calculated osmolarity and bedside assessment in older hospitalised patients. We found that glucose concentrations were higher and more variable in the osmolarity-positive groups (false positives and true positives) than in osmolarity-negative groups, and pairwise comparisons demonstrated significantly higher mean glucose in false positives versus true negatives and in true positives versus false negatives. This pattern suggests that hyperglycaemia frequently co-occurred with elevated calculated osmolarity and may have contributed to discordant dehydration classification in some patients. In older hospitalised patients, where hyperglycaemia is common, calculated osmolarity may therefore be particularly susceptible to “glucose-driven” elevations.

Consistent with this, the diagnostic profile observed in our study showed poor sensitivity and specificity, with a particularly low positive predictive value, indicating that a substantial proportion of patients with calculated osmolarity ≥ 295 mOsmol/l were not clinically judged dehydrated. Our findings are consistent with the small hospital study by McCrow et al., which also compared clinical dehydration assessment with calculated osmolarity (≥ 295 mOsmol/l) [25]. In that study, clinicians classified 27% of patients as dehydrated at admission compared with 19% by calculated osmolarity, and agreement between the two approaches was only fair at admission and poor at study exit. Using calculated osmolarity as the comparator, the authors reported low sensitivity of clinical assessment with moderate specificity (sensitivity 0.50 and specificity 0.77 at admission). Similar observations were reported by Deissler and colleagues; although formal diagnostic accuracy estimates were not provided since osmolarity did not differ significantly between patients classified as hypohydrated, normally hydrated, or hyperhydrated [24]. Taken together with our results, these data support that clinical impression and calculated osmolarity frequently classify different patients as dehydrated.

Therefore, these results suggest that calculated osmolarity ≥ 295 mOsmol/l should be interpreted cautiously and not as a stand-alone indicator of clinical dehydration in acutely older hospitalised patients. In settings where abnormalities in glucose and urea are common, relying on calculated osmolarity using formulas that include glucose, and, to some extent, even on directly measured osmolality, may lead to substantial misclassification of hydration status. In addition, a relevant proportion of clinically dehydrated patients, particularly in acute care, may suffer from other forms of dehydration, such as isotonic and hypotonic dehydration, as a potential explanation for the low diagnostic performance of calculated osmolarity. Moreover, another explanation for the limited agreement is that clinical impression may detect signs of extracellular volume depletion and water loss, whereas calculated osmolarity mainly reflects hypertonic low-intake dehydration, therefore the two approaches may not identify the same physiological condition [11, 26]. Future studies should examine whether alternative cut-offs, formulas without glucose, glucose-based adjustments, or combined clinical and laboratory approaches improve dehydration assessment in older hospitalised patients. These approaches should ideally be compared with directly measured osmolality and validated in an independent cohort.

This study has several limitations that should be acknowledged. First, clinical impression was used as the reference standard and, although it reflects real-world practice, it is not a fully standardized or validated gold standard for dehydration in older adults and may vary between physicians. Therefore, these diagnostic measures should be interpreted in relation to the clinical impression used in this study, rather than as measures of accuracy against a true gold standard. In addition, no standardized diagnostic score was used and inter-rater reliability between physicians was not assessed in our study, which may have introduced variability in the clinical classification. However, in this study the assessment was performed by experienced geriatricians within 24 h of admission and was based on an integrated evaluation of all available routine information, including anamnesis, physical examination (e.g., mucosal/skin dryness, venous filling, oedema), comorbidities, medication, laboratory parameters, and, when available, ultrasound assessment of the inferior vena cava. Second, the cross-sectional and single-center design may limit the generalisability of our results. Third, acute illness and comorbidities common in older inpatients (e.g., hyperglycaemia and renal dysfunction) may influence calculated osmolarity independently of hydration status and could contribute to misclassification. In addition, while calculated serum osmolarity is pragmatic and widely available, direct measurement of osmolality would have been preferable but was not feasible in this study. Finally, patients with severe disturbances of fluid balance were excluded according to the original study design aiming for malnutrition, which may have reduced the number of patients with more severe dehydration and weakened the observed differences between dehydrated and non-dehydrated groups.

A major strength is the large, clinically relevant cohort of older hospitalised patients, allowing a real-world evaluation of calculated osmolarity against bedside clinical assessment, supported by standardised blood sampling early during admission. Future studies should assess whether alternative cut-offs and/or modified calculation approaches (for example formula without glucose) improve alignment with clinically meaningful dehydration in hospitalised older adults.

## Conclusion

In older hospitalised patients, calculated serum osmolarity using the commonly applied cut-off of ≥ 295 mOsmol/l showed limited agreement with clinical impression of dehydration, with many osmolarity-positive classifications occurring in patients not judged clinically dehydrated. These findings indicate that calculated osmolarity should be interpreted cautiously as a stand-alone marker of dehydration in older hospitalised patients, and confirmatory assessment and/or refined approaches may be needed in routine hospital practice.

## Supplementary information

Below is the link to the electronic supplementary material.

Supplementary Material 1

## Data Availability

No datasets were generated or analysed during the current study.

## Declarations

### Availability of data and materials

The datasets analyzed during the current study are available from the corresponding author on reasonable request.
