# Supplementary material for: Diagnostic performance of calculated serum osmolarity versus clinical impression for identifying dehydration in older hospitalized patients
Source: BMC Geriatr. 2026 Oct 16;26:1374. doi: 10.1186/s12877-026-08503-6 (PMC13726267; doi:10.1186/s12877-026-08503-6)
Supplement: Supplementary file 1 [file 12877_2026_8503_MOESM1_ESM.docx]

| **Supplementary File 1. STROBE checklist for cross-sectional studies** | | | |
| --- | --- | --- | --- |
| **STROBE section / item** | **Item No.** | **Recommendation** | **Reported on page no.** |
| **Title and abstract – Study design** | 1(a) | Indicate the study design with a commonly used term in the title or abstract | Abstract, p. 2, lines 31–32; Methods, Section 2.1, p. 5, lines 116–127 |
| **Title and abstract – Abstract** | 1(b) | Provide an informative and balanced summary of what was done and what was found | Abstract, p. 2, lines 26–52 |
| **Introduction – Background/rationale** | 2 | Explain the scientific background and rationale for the investigation being reported | Introduction, Section 1, pp. 3–4, lines 54–107 |
| **Introduction – Objectives** | 3 | State specific objectives, including any prespecified hypotheses | Introduction, Section 1, p. 4, lines 108–114 |
| **Methods – Study design** | 4 | Present key elements of study design early in the paper | Methods, Section 2.1, p. 5, lines 116–127 |
| **Methods – Setting** | 5 | Describe the setting, locations, and relevant dates, including periods of recruitment and data collection | Methods, Section 2.1, p. 5, lines 117–133 |
| **Methods – Participants** | 6(a) | Give eligibility criteria and the sources and methods of selection of participants | Methods, Section 2.1, p. 5, lines 128–133 |
| **Methods – Variables** | 7 | Clearly define outcomes, exposures, predictors, potential confounders, and diagnostic criteria, if applicable | Methods, Sections 2.2–2.3, pp. 5–7, lines 134–185 |
| **Methods – Data sources/measurement** | 8* | For each variable of interest, give sources of data and details of methods of assessment/measurement | Methods, Sections 2.2–2.3, pp. 5–7, lines 134–185 |
| **Methods – Bias** | 9 | Describe any efforts to address potential sources of bias | Methods, Section 2.3, pp. 6–7, lines 151–185; Discussion, Limitations, p. 12, lines 330–350 |
| **Methods – Study size** | 10 | Explain how the study size was arrived at | Methods, Section 2.1, p. 5, lines 125–127 |
| **Methods – Quantitative variables** | 11 | Explain how quantitative variables were handled in the analyses; if applicable, describe which groupings were chosen and why | Methods, Section 2.3, pp. 6–7, lines 161–176; Statistical methods, Section 3, pp. 7–8, lines 186–206 |
| **Methods – Statistical methods** | 12(a) | Describe all statistical methods, including those used to control for confounding | Statistical methods, Section 3, pp. 7–8, lines 186–206 |
| **Methods – Subgroups/interactions** | 12(b) | Describe any methods used to examine subgroups and interactions | Results, Section 4.4, p. 9, lines 245–252; Figure 2 legend, p. 16, lines 469–476 |
| **Methods – Missing data** | 12(c) | Explain how missing data were addressed | Not explicitly reported |
| **Methods – Sampling strategy** | 12(d) | If applicable, describe analytical methods taking account of sampling strategy | Not applicable |
| **Methods – Sensitivity analyses** | 12(e) | Describe any sensitivity analyses | Not applicable |
| **Results – Participants** | 13(a)* | Report numbers of individuals at each stage of the study | Methods, Section 2.1, p. 5, lines 128–133; Results, Section 4.1, p. 8, lines 209–214; Tables 1–3, pp. 17–19 |
| **Results – Non-participation** | 13(b)* | Give reasons for non-participation at each stage | Methods, Section 2.1, p. 5, lines 128–133 |
| **Results – Flow diagram** | 13(c)* | Consider use of a flow diagram | Not provided |
| **Results – Descriptive data** | 14(a)* | Give characteristics of study participants and information on relevant exposures and potential confounders | Results, Sections 4.1–4.2, p. 8, lines 209–228; Tables 1–2, pp. 17–18 |
| **Results – Missing data** | 14(b)* | Indicate number of participants with missing data for each variable of interest | Results, Section 4.1, p. 8, lines 209–214; Tables 1–3, pp. 17–19 |
| **Results – Outcome data** | 15* | Report numbers of outcome events or summary measures | Results, Section 4.1, p. 8, lines 209–214; Section 4.3, pp. 8–9, lines 229–244; Table 3, p. 19 |
| **Results – Main results / estimates** | 16(a) | Give unadjusted estimates and, if applicable, adjusted estimates and their precision | Results, Section 4.3, pp. 8–9, lines 229–244; Table 3, p. 19 |
| **Results – Category boundaries** | 16(b) | Report category boundaries when continuous variables were categorized | Methods, Section 2.3, pp. 6–7, lines 161–176; Table 3, p. 19 |
| **Results – Absolute risk** | 16(c) | If relevant, translate estimates of relative risk into absolute risk | Not applicable |
| **Results – Other analyses** | 17 | Report other analyses performed, such as subgroup, interaction, or sensitivity analyses | Results, Sections 4.3–4.4, pp. 8–9, lines 229–252; Figure legends, p. 16, lines 466–476 |
| **Discussion – Key results** | 18 | Summarize key results with reference to study objectives | Discussion, Section 5, pp. 9–12, lines 253–356 |
| **Discussion – Limitations** | 19 | Discuss limitations of the study, taking into account potential sources of bias or imprecision | Discussion, Limitations, p. 12, lines 330–350 |
| **Discussion – Interpretation** | 20 | Give a cautious overall interpretation considering objectives, limitations, multiplicity of analyses, similar studies, and other relevant evidence | Discussion, Section 5, pp. 9–12, lines 253–356 |
| **Discussion – Generalisability** | 21 | Discuss the generalisability (external validity) of the study results | Discussion, Limitations, p. 12, lines 330–350 |
| **Other information – Funding** | 22 | Give the source of funding and the role of funders for the present study and, if applicable, the original study | Declarations – Funding, p. 13, lines 378–379 |
